# The heterogeneity within Alzheimer's disease

**DOI:** 10.18632/aging.101638

**Published:** 2018-11-14

**Authors:** Daniel Ferreira, Lars-Olof Wahlund, Eric Westman

**Affiliations:** 1 Division of Clinical Geriatrics, Centre for Alzheimer Research, Department of Neurobiology, Care Sciences and Society, Karolinska Institutet, Stockholm, Sweden; 2 Department of Neuroimaging; Centre for Neuroimaging Sciences; Institute of Psychiatry, Psychology, and Neuroscience, King’s College London, London, UK

**Keywords:** Alzheimer’s disease, subtypes, heterogeneity, cerebrovascular disease, cognitive reserve, clinical trials

During the last decade, we have experienced a difficult period with over 200 failed clinical trials and still no cure for Alzheimer’s disease (AD). The scientific community has been critical regarding this outcome and a few potential explanations have been discussed. Firstly, the recruited populations have often been heterogeneous. Secondly, patients were demented and although mildly to moderately, most presented with irreversible brain damage, thus jeopardizing the potential effect of the drugs. Thirdly, we may have been aiming at the wrong targets, or at least we may have tried to simplify a multifactorial disease that may never be defeated with a magic bullet.

We need to review the evidence indicating both clinical and pathophysiological heterogeneity in AD. For instance, tangle pathology has long been thought to spread across the brain in a typical manner, initiating in the transentorhinal cortex, then spreading to the entorhinal and hippocampal areas, and finally extending to the lateral association cortex. This pattern would mostly underlie the typical amnestic presentation of AD. However, other subtypes have also been described histopathologically in which tau mainly affects the hippocampus (limbic-predominant AD) or predominantly occupies the association cortex (hippocampal-sparing AD) [1]. In this regard, the hippocampal-sparing subtype is more frequently related to non-amnestic presentations of AD, as well as to non-AD clinical diagnoses [1,2]. This differential spread of tangle pathology can be reliably tracked in vivo with the help of magnetic resonance imaging (MRI) [2]. Recent studies have also identified a fourth AD subtype that displays minimal brain atrophy with similar disease severity as compared to the other subtypes [3].

In addition, postmortem data clearly shows that AD pathology rarely occurs in isolation [4]. Most AD patients harbor more than one pathology in the brain, with cerebrovascular disease being the most common coexisting pathology. Furthermore, the frequency of both cerebrovascular and Alzheimer’s disease increases with age. However, in what way cerebrovascular disease and AD pathology act in synergy leading to downstream neurodegeneration and dementia is still unknown. Cerebral amyloid angiopathy (CAA), a form of cerebrovascular disease resulting from amyloid deposition in vessel walls, may be the link between these two frequently coexisting pathologies. It is interesting that anti-amyloid therapy has been reported to increase the incidence of microbleeds, potentially due to removal of amyloid through vessel walls. The big question is whether CAA is just a passenger on the AD train. How does CAA interact with amyloid and tau pathology? For instance, does CAA come in early on in disease pathogenesis by affecting the spread of neurofibrillary tangles across the brain? Or is CAA an event occurring in later stages, acting downstream to amyloid and tau pathology thus mostly contributing to neurodegeneration and brain atrophy? All these questions remain largely unanswered.

**Figure 1:**
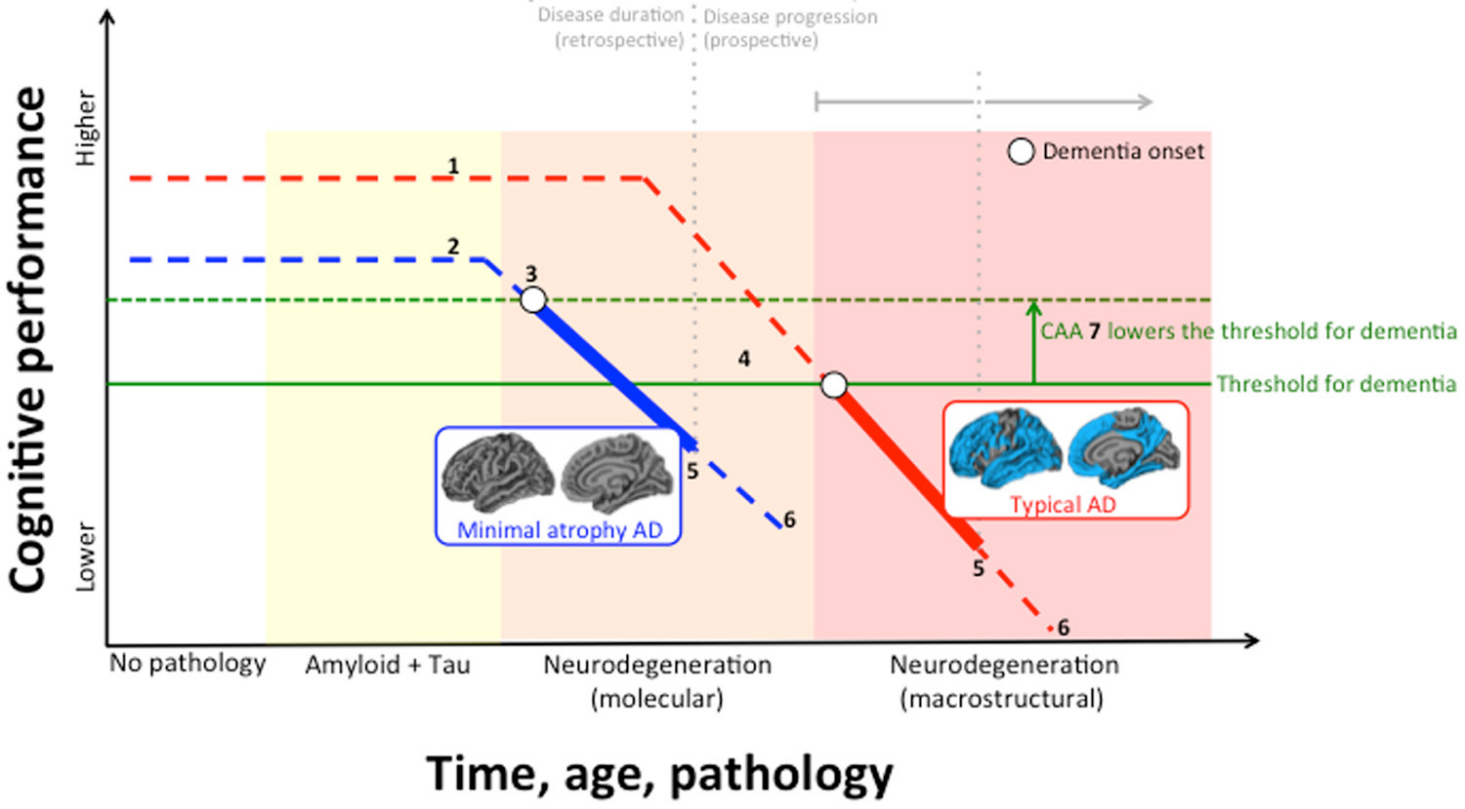
**Hypothetical interrelation between subtypes of Alzheimer’s disease (AD), neurodegeneration, cerebral amyloid angiopathy (CAA), and cognitive reserve.** (**1**) Higher cognitive reserve; (**2**) Lower cognitive reserve; (**3**) Earlier disease onset in minimal atrophy AD (disease onset in the figure refers to first symptoms of dementia, not to first evidence for biomarker abnormality); (**4**) Later disease onset in typical AD; (**5**) Similar disease duration at baseline (retrospective) between typical and minimal atrophy AD, and similar cognitive performance at baseline, with typical AD usually showing slightly more impairment; (**6**) Faster disease progression (prospective) with typical AD reaching severe dementia in shorter time; (**7**) CAA is more frequent in minimal atrophy AD and may lower the threshold for the amount of AD pathology needed to produce dementia.

We recently conducted a comprehensive characterization of these AD subtypes in terms of cerebrovascular disease, including the amount and distribution of deep/lobar microbleeds and white matter hyperintensities, cortical superficial siderosis, perivascular spaces, lacunes, large brain infarction, and intracerebral hemorrhage [5]. We concluded that CAA seems to make a stronger contribution to hippocampal-sparing and minimal atrophy AD, whereas hypertensive arteriopathy, another form of cerebrovascular disease, may make a stronger contribution to typical and limbic-predominant AD. This study also revealed important mechanisms for minimal atrophy AD patients who harbor pathological levels of amyloid, tau, and neurodegeneration biomarkers in their cerebrospinal fluid, but who do not display overt brain atrophy. We speculate that due to low cognitive reserve in this subtype, neurodegeneration at the molecular level (i.e. cerebrospinal fluid) is sufficient to produce clinical symptoms of AD in the absence of neurodegeneration at the macrostructural level (i.e. MRI). Indeed, the finding of low cognitive reserve in minimal atrophy AD has been observed in several independent cohorts [5,6]. Since we found that increased CAA is associated with lower cognitive performance in minimal atrophy AD [5], CAA in this subtype could contribute to lowering the threshold for the amount of AD pathology needed to produce cognitive impairment and dementia. Nonetheless, AD patients with minimal atrophy have progressed enough to display similar clinical severity compared to the other three subtypes. Of relevance, minimal atrophy AD patients are rather comparable in disease duration but they have slower cognitive decline over time [3]. These findings, together with the fact that they are usually younger, make minimal atrophy AD a good candidate for future intervention studies. Lower cognitive reserve leaves room for building up late-life cognitive resilience by increasing engagement in cognitive activities and exposure to leisure or physical activities. From a pharmacological perspective, it would be interesting to investigate how well the minimal atrophy subtype responds to acetylcholinesterase inhibitors. Previous studies have shown that patients with preserved medial temporal lobes respond better to that treatment [7]. The Figure 1 below shows how CAA and cognitive reserve may hypothetically modulate disease progression, which is thought to be primarily caused by the accumulative onslaught of amyloid, tau, and neurodegenerative pathology.

Evidence suggests that neurodegeneration can be expressed differently across different AD subtypes. Future research will also have to answer why amyloid pathology starts, what is triggering the cascade, and whether this differs in the different subtypes. Current data shows that dementia in AD is a downstream event that can be reached along different pathways. These different pathways may necessitate their own specific therapeutic strategies. The differences in biological factors (and modifiable life factors) between these AD subtypes may guide the potential targets for future trials. Recognizing the heterogeneity within AD implies opening the door to multifactorial intervention strategies. We believe that unraveling the heterogeneity within AD can promote personalized medicine approaches in the short term by guiding tailored cognitive interventions, and help in characterizing more homogeneous AD groups for drug discovery in the future. This may facilitate the tough endeavor of finding a cure for this disease.
